# Mitochondrial Toxicity in Human Pregnancy: An Update on Clinical and Experimental Approaches in the Last 10 Years

**DOI:** 10.3390/ijerph110909897

**Published:** 2014-09-22

**Authors:** Constanza Morén, Sandra Hernández, Mariona Guitart-Mampel, Glòria Garrabou

**Affiliations:** 1 Muscle Research and Mitochondrial Function Laboratory, Cellex-IDIBAPS-Faculty of Medicine-University of Barcelona, Internal Medicine Service-Hospital Clínic of Barcelona, Barcelona 08036, Spain; E-Mail: garrabou@clinic.ub.es; 2 Centro de Investigación Biomédica en Red (CIBER) de Enfermedades Raras, CIBERER, Valencia 46010, Spain; E-Mail: ashernan@clinic.ub.es; 3 Materno-Fetal Medicine Department, Clinical Institute of Gynaecology, Obstetrics and Neonatology, Barcelona 08025, Spain

**Keywords:** Mitochondria, toxic effects, pregnancy, adverse obstetric outcome

## Abstract

Mitochondrial toxicity can be one of the most dreadful consequences of exposure to a wide range of external agents including pathogens, therapeutic agents, abuse drugs, toxic gases and other harmful chemical substances. However, little is known about the effects of mitochondrial toxicity on pregnant women exposed to these agents that may exert transplacental activity and condition fetal remodeling. It has been hypothesized that mitochondrial toxicity may be involved in some adverse obstetric outcomes. In the present study, we investigated the association between exposure to mitochondrial toxic agents and pathologic conditions ranging from fertility defects, detrimental fetal development and impaired newborn health due to intra-uterine exposure. We have reviewed data from studies in human subjects to propose mechanisms of mitochondrial toxicity that could be associated with the symptoms present in both exposed pregnant and fetal patients. Since some therapeutic interventions or accidental exposure cannot be avoided, further research is needed to gain insight into the molecular pathways leading to mitochondrial toxicity during pregnancy. The ultimate objective of these studies should be to reduce the mitochondrial toxicity of these agents and establish biomarkers for gestational monitoring of harmful effects.

## 1. Introduction

### 1.1. Mitochondrial Physiology

Mitochondria are cell organelles located in the cytoplasm of most of the eukaryotic cells [1]. Mitochondria are essential for cell viability because of their involvement in many important processes, such as heat production, energy supply, cell respiration, calcium homeostasis and the anabolism and catabolism of numerous metabolites [2], among others. However, in pathological conditions, mitochondria are also the main source of reactive oxygen species (ROS) production and apoptosis.

These organelles are the energy powerhouse of cells as they provide energy through the formation of molecules of adenosine triphosphate (ATP), the major energy source of the cells. The synthesis of ATP is coupled to cell respiration and oxygen consumption in the mitochondrial respiratory chain (MRC).

Mitochondria are constituted by a double membrane; the external membrane, which is permeable to many solutes and makes the interchange of molecules with the cytosol possible, and the internal membrane, which is highly impermeable and is a folded structure constituting the mitochondrial cristae, where the enzymatic complexes of the MRC are located. Together with chloroplasts, mitochondria are the only organelles containing their own genetic material, the mitochondrial DNA (mtDNA) encoding for some proteins of the MRC and autonomous transcriptional and translational machinery. Human mtDNA is a 16.6 kb double-stranded circular and covalently closed molecule encoding for 13 MRC proteins, two mitochondrial ribosomal RNA and 22 mitochondrial transfer RNA essential for the translation of mitochondrial-encoded proteins [3]. The remaining proteins located in the mitochondria (approximately 1500 in mammals) are encoded in the nucleus. Consequently, intergenomic communication between these two entities is essential for adequate mitochondrial function. In this context, a decrease of mtDNA levels (mtDNA depletion) can ultimately lead to severe mitochondrial dysfunction and energetic cell impairment. Additionally, mitochondrial disarrangements can arise during many other steps of mitochondrial function (biogenesis, bioenergetics, dynamics or turnover), independently of mtDNA depletion.

Mitochondrial DNA is exclusively transmitted by the maternal lineage, contrarily to nuclear DNA that is transmitted by both parents, and the variation in number of both mitochondria and mtDNA content varies widely depending on the cell type and stage of development. Somatic cells contain from hundreds to thousands of mitochondria, each carrying from 2 to 10 genomes per organelle [3], and postmitotic tissues, which are highly-energetic and dependent on oxidative metabolism, present higher loads of mitochondria and mtDNA content. Accordingly, a physiologic state associated with a high energy demand, such as fertilization and pregnancy, may require greater mitochondrial activity.

The oocyte is the only reproductive cell that provides mitochondrial load to the future embryo and posterior newborn. Thus, mitochondria are maternally inherited and a sufficient energy supply from oocyte mitochondria is critical to trigger oocyte viability and future embryo development. The oocyte is the largest human cell (on average 300 times bigger than other somatic cells) and contains a large amount of mitochondria that represent at least 23% of the ooplasm [4]. Depending on the stage of development of the germinal cells, the number of mitochondria present ranges widely from 10 in germinal cells, 1000 in blastocytes to 100,000 in mature oocytes. Oocytes are packed with mitochondria, each of which has its own genome. The mtDNA copy number per mature human oocyte is about 100,000–600,000 molecules, compared with the 500–10,000 molecules for the remaining somatic cells [5,6]. However, this very large number of mtDNA per oocyte is not due to increased mtDNA content per mitochondria but rather to an increased number of mitochondria per oocyte. In fact, along oogenesis, the number of copies of mtDNA is thought to be reduced per organelle in a process known as the mitochondrial bottleneck. The aim of this evolutionary strategy is to finally contain one mtDNA molecule per mitochondrion to avoid heteroplasmic segregation through the maternal lineage [7], thereby avoiding the coexistence of wild type and mutated molecules of mtDNA within the same entity (mitochondria, oocyte and future embryo). However, this makes oocyte mitochondria especially vulnerable to mtDNA depletion and also makes oocytes especially sensitive to factors causing mtDNA depletion or mutagenesis, such as infections, drugs and toxic substances, among others.

The involvement of mitochondria in fertility outcome can be easily estimated *in vitro*. It has been reported that 50% of human *in vitro* fertilization (IVF) attempts fail during the first week of development [8]. The main causes of this developmental failure, apart from chromosomic alterations [9] (deficiency in oocyte maturation [10], lack of activation of embryonic genome [11] or sub-optimal culture conditions of both gametes and embryos [12,13]) have been directly or indirectly related to mitochondrial function and ROS production. Cohen *et al*. reported that ooplasm transfer from a young donor oocyte into a non-fertile oocyte partially restores the reproductive capacity in the oopausic oocyte, and they suggested that “fertility” restoration may be due to mitochondrial transference [14]. Other studies have shown that both mitochondrial and mtDNA content reflect oocyte variability and fertilization outcome [4]. Thus, *in vitro* studies have suggested that mitochondria in the oocyte contribute to successful fertilization and embryonic development.

Although spermatozoa do not provide mitochondria to the future embryo, mitochondrial function is essential for flagellum motility and male fertility in the spermatozoa.

Once fertilization and implantation have been achieved, mitochondrial activity is essential along pregnancy to provide energy and metabolites for the development of the embryo. Blastocytes and future embryo cells need an enormous supply of energy to feed constant cell division, migration and differentiation. Additionally, mitochondria regulate apoptosis development in this crucial stage of life to decide which cells or tissues need to be eliminated for further embryo development. Consequently, exposure of mitochondria to toxic agents during pregnancy can alter the development of the embryo and may have important consequences in the perinatal outcome and health of the newborn.

### 1.2. Mitochondrial Pathology

Mitochondria are essential for life and the presence of mitochondrial alterations in a given organism may lead to the development of mitochondriopathies [15]. Mitochondrial diseases are classified as inherited or acquired (derived from toxic substances), with both sharing similar clinical consequences.

Mitochondria are present in almost all the tissues of the organism, and therefore mitochondrial diseases can be translated into a wide spectrum of clinical manifestations [16]. Mitochondrial diseases lack therapeutic treatments and are characterized by the degeneration of tissues, especially those which are highly energetic such as muscle and nervous tissue. The symptoms range from myopathy, neuropathy, encephalopathy, lactic acidosis, to lipodystrophy or deafness [15]. In the context of pregnancy, mitochondrial dysfunction has been associated with increased rates of preterm delivery, stillbirth, intrauterine growth restriction (IUGR), and sudden infant death [17].

The mutations responsible for genetic mitochondrial diseases can be present in both the nuclear or mitochondrial genome. Genetic mitochondrial diseases are present in 1 in 5000 newborns and 1 in 200 women may carry one of these deleterious mutations. Nuclear mutations are paternal and maternally inherited due to Mendelian heritance and can be prevented through genetic counseling and pre-implantational diagnosis. However, inherited or acquired mutations in mtDNA present a random maternal inheritance pattern due to heteroplasmy that hampers both the diagnosis and prevention of mitochondrial diseases.

Genetically inherited mitochondrial diseases affect the offspring of carriers of nuclear or mtDNA mutations. However, acquired mitochondrial diseases are potentially caused by exposure to multiple exogenous factors such as: biologic agents, therapeutic drugs, abuse drugs, toxic gases and chemical substances, regardless of the genetic environment.

These toxic compounds usually exert their damaging effects by impairing a specific genetic, biochemical or molecular mitochondrial pathway [18]. Nonetheless, in cases of chronic abuse, most of these toxic agents finally lead to general mitochondrial dysfunction which can compromise cellular and tissue viability and, in some cases, be life threatening [17]. Mitochondrial recovery may occur once the exogenous toxicant is withdrawn [19], and the clinical effects caused by these agents normally disappear with discontinuation of exposure. However, when this is not possible, clinicians must manage the secondary effects caused by mitochondrial toxicity. Mitochondrial therapies designed to revert mitochondrial-induced damage (such as antioxidants or vitamins, among others) are currently being developed but, are not yet available in routine clinical practice, especially for the management of pregnancy or newborns. These treatments involve symptomatic and supportive therapies. Thus, to date, the prevention of mitochondrial acquired diseases and mitochondrial toxicity is the prophylactic therapeutic option of choice [20].

Although mitochondrial toxicity has been widely studied in adults, there is relatively little information on these toxicities within the context of human pregnancy, particularly *intra utero* exposure and the potential impact on fetal development and the future of the newborn. The objective of this work was, therefore, to review mitochondrial toxicity in fertility and human pregnancies.

## 2. Methods

We reviewed all the literature concerning fertilization, obstetric and perinatal outcomes due to exposure to any mitochondrial toxic agent in humans as an update on the studies performed in these areas in the last 10 years.

We performed a systematic review through PubMed/MEDLINE using keyword search terms related to mitochondrial toxic agents, in the English language, and involving human participants.

The literature review included all the articles published in peer-review journals related to data on mitochondrial toxicity in pregnancies exposed to toxic compounds, following the same model of mitochondrial studies of toxicities derived from exposure to determined toxic agents described in non-pregnant adults. The literature review was conducted with searches in PubMed using combinations of the fixed medical subject headings (MeSH): pregnancy and mitochondria, together with the variable headings, related to poisonous exposure agents. A significant amount of literature was found with the association of mitochondria and pregnancies. However, in most cases, the number of documents available considerably decreased when a third factor, related to the toxic agent, was added to the search.

Except for some punctual cases, we restricted our review to material published in the last 10 years (2004–2014) to obtain the most recent information in this field.

All English language articles with a full-text version reporting data about mitochondrial toxicity in human pregnancies exposed to toxic agents with potential capability of causing mitochondrial alterations were included in the review. Articles lacking an English abstract were excluded. The references of these articles were also scanned for potential additional material, but this did not yield many studies fulfilling the study criteria. Studies including animal models or information of highly specific basic sciences were excluded from the present review, except for those describing the mechanism of toxicity of a studied agent or those needed to reinforce the strong need for further research in human pregnancies due to extensive evidence of toxicity in experimental models.

We found very few studies describing an association between mitochondrial toxicity and human pregnancy or reproductive outcome with respect to fertilization or fetal development. Nonetheless, we have summarized the information available regarding exposure to: biologic agents (human immunodeficiency virus, HIV and hepatitis C virus, HCV), drugs (antivirals, antipsychotics, antibiotics, hypolipemia drugs, antidiabetics, non-steroidal anti-inflammatory drugs (NSAIDs), anaesthetics, chemotherapy drugs, antiarrhythmics, antimalarials and fungicides), abuse drugs (such as tobacco and alcohol), toxic gases (as carbon monoxide) and chemical substances (including pesticides).

## 3. Results

### 3.1. Mitochondrial Toxicity of Biologic Agents during Pregnancy; The Effect of HCV or HIV

Both HCV and/or HIV-pregnancies have been associated with an increased frequency of adverse obstetric and perinatal outcomes both of which result in maternal or neonatal complications [21,22].

The HCV has recently been related to an increase of oxidative stress and mtDNA alterations in non-pregnant infected patients. However, to our knowledge, scarce information is available on HCV-infected pregnant women to assess viral-mediated mitochondrial damage and its association with adverse perinatal outcomes [23].

The mechanisms underlying vertical transmission of HCV are poorly understood. Intrauterine transmission during pregnancy and infection at the time of delivery are both possible. To date, no special measures are taken for HCV-pregnancies to avoid mother-to-child transmission (MTCT).

On the other hand, in non-pregnant patients, HIV is known to cause diffuse mitochondrial impairment by promoting cell death through apoptosis triggered by certain viral proteins [24]. Mitochondrial alterations associated with HIV itself were first described in 2002 [25] in a study in which mtDNA depletion was reported in peripheral blood mononuclear cells (PBMC) of HIV-patients who had never received antiretroviral (ARV) therapy [25]. Thereafter, mitochondrial dysfunction was also demonstrated in naïve patients [26]. However, only a few studies have analyzed the toxic mitochondrial effects of HIV-infection in human pregnancies.

The exact mechanism of MTCT of HIV remains unknown. Transmission may occur during intrauterine life, delivery, or breastfeeding. Advanced maternal disease, probably due to high viral load, is the greatest risk factor for vertical transmission. Untreated HIV-pregnancies are associated with high transmission rates of up to 25–30%. With the implementation of universal prenatal HIV-testing, counseling, ARV medication, delivery by cesarean section prior to the onset of labor, and avoidance of breastfeeding, MTCT has decreased to less than 1–2% in developed countries [27,28]. The administration of ARV to avoid HIV-MTCT has reduced viral burdens at delivery and, consequently, viral-mediated mitochondrial damage should be minimal [29]. However, some antiviral drugs have been shown to cause secondary mitochondrial toxicity. These adverse effects during pregnancy are reviewed below in the section on “Drugs”. Most of the studies reporting mitochondrial damage in perinatal HIV-exposed newborns include information about transplacentally ARV-derived adverse effects, rather than describe the effects of the virus itself, regardless of the therapy used. However, the inflammatory environment created by both the HCV and HIV (pro-inflammatory cytokine expression or humoral and cellular immune activation, among others) [30] may also reduce the fertility and pregnancy outcome of infected women independently of ARV. Thus, further research on mitochondrial toxicity due to viral infection during pregnancy is required for better understanding of mitochondrial implications in obstetric outcomes in these pregnancies.

### 3.2. Drugs

#### 3.2.1. Antivirals

Concerning antivirals against HIV, international guidelines recommend the administration of ARV, at least during the last trimester of gestation, to decrease the viral load at delivery and the risk of MTCT.

The potential clinical risks associated with ARV exposure in HIV-pregnant women, fetuses and infants have been described in observational studies with varying degrees of evidence and conflicting results [31,32,33,34,35]. Antiretroviral drugs are essential in the treatment and prevention of HIV-infection and transmission. Although their use before, during and after pregnancy is considered safe for both the mother and child, there are still lingering concerns about their long-term health consequences and the ramifications of their effects on lipid, glucose, intermediary and mitochondrial metabolism [36]. Additionally, ARV have been associated with adverse pregnancy outcomes such as preeclampsia, stillbirth, preterm birth and low birth weight, although controversial results have been published [37,38,39,40,41].

Antiretroviral-driven mitochondrial toxicity has been especially associated with the administration of nucleoside analogs which are known to inhibit the mitochondrial enzyme responsible for mtDNA replication and repair (DNA polymerase gamma), reviewed in [42]. Nucleoside analog treatment has been widely associated with mtDNA point mutations, deletions and depletion responsible for adverse manifestations in treated patients including lactic acidosis, lipodystrophy as well as infertility [43]. Infertile HIV-infected women on highly active ARV therapy showed oocyte mtDNA depletion of 32%, which was even greater in HIV-infected women who failed to become pregnant after IVF [44]. The authors of this study did not find any correlation between mtDNA oocyte content and the immunovirological status of pregnant women. Additionally, oocytes have no HIV-receptors for viral entrance, suggesting that the alterations detected may be due to drug toxicity rather than viral infection. The mitochondrial toxicity of ARV may trigger the impairment of female fertility but, additionally, when pregnancy is achieved, mitochondrial toxicity may increase adverse human pregnancy outcomes. Negative mitochondrial and clinical effects of ARV therapy have been reported in HIV-pregnancies [45].

Most of the mitochondrial studies performed in HIV-pregnancies have described an increased frequency of adverse obstetric events in the HIV-cohort [45,46,47,48,49]. Some controversial results in HIV-exposed infants reported increased levels of fetal leukocyte mtDNA content [50] accompanied by reduced umbilical cord blood mitochondrial enzyme expression, leading the authors to hypothesize a compensatory mechanism to overcome HIV/ARV-associated mitochondrial toxicity [50]. However, most of the studies reported different degrees of evidence of increased mitochondrial toxicity and, in some cases, the development of apoptosis in maternal, fetal or even placental tissue [45,46,47,48,49]. The small sample size of these studies makes it difficult to link mitochondrial toxicity or the development of apoptosis with adverse pregnancy outcomes because of the reduced statistical power when classifying the HIV-cohort according to successful pregnancy results. Further studies with larger sample sizes are required.

#### 3.2.2. Antipsychotics, Antibiotics, Hypolipemia Drugs and Antidiabetics, Non-Steroidal Anti-inflammatory Drugs (NSAIDs), Anesthetics, Chemotherapy Drugs, Antiarrhythmics, Antimalarials and Fungicides

Similarly, many other drugs currently used in clinical practice as acute treatments in non-pregnant subjects have been described to cause mitochondrial toxicity by impairing different pathways of mitochondrial function. Mitochondrial respiratory chain function has been reported to be blocked by some antibiotics (including piericidin A or antimycin A) [51], certain anaesthetic drugs [52] or some barbiturates [53], antineoplastic treatments (including flutamide, tamoxifen and doxorubicin) [54,55] and fungicidal agents (such as myxothiazole, sodium azide or rutamycin) [56]. Some of these agents may lead to the impairment of cell respiration (fibrates) [57]. Other drugs can act as uncouplers of the oxidative phosphorylation system (antibiotics such as valinomycin and gramicidin, local anaesthetics and antineoplastics including flutamide, tamoxifen and doxorubicin) [55]. Likewise, other drugs may prevent ATP production (antibiotics such as oligomycin or local anaesthetic drugs), inhibit protein synthesis (antibiotics including chloramphenicol, tetracycline, erythromycin, eperezolid, linezolid and aminoglycosides) [19,58], decrease electron transfer (anticholesterolemics) or membrane potential, impair mitochondrial lipid metabolic pathways (barbiturates) or increase apoptosis (including benzodiazepines) [59]. Such mitochondrial disturbances have been reported to cause adverse effects including deafness [60], peripheral neuropathy [61], hyperlacatemia, lactic acidosis [62] and gastrointestinal, dermatological or hematological alterations, myopathic syndrome or gastrointestinal disturbances, among others [63]. Specific safety and mitochondrial toxic studies in human pregnancies are lacking for most of these drugs. Indeed, most of these treatments are contraindicated in pregnancy (for instance, chloramphenicol, tetracyclines, linezolid, most aminoglycosides, oral hypolipemiadrugs or fungicides). However, during pregnancy, women could be exposed to these substances in some exceptional cases: when they are not aware of their pregnancy status, as punctual and short interventions in case of emergency (for instance erythromycin) or as mandatory treatments for chronic diseases, among others.

This is the case of drugs used for mental health disorders such as schizophrenia, bipolar disorder, and psychotic depression, which are not rare in women of childbearing age. Similar to ARV, antipsychotic drugs in the management of antenatal psychiatric disorders are not of choice but are strongly recommended or even mandatory in psychiatric patients during pregnancy. Antipsychotics are associated with increased gestational weight and diabetes and increased risk of preterm birth. The effects of antipsychotics on low birth weight or malformations are inconclusive. From a mitochondrial point of view, antipsychotics have been described to inhibit MRC complex I function, causing an increase in the levels of oxidative stress in non-pregnant adults [64]. However, no study was found regarding the analysis of mitochondrial toxicity of neuroleptic drugs in human pregnancies. 

On the other hand, although the hypoglycemiant drugs are not administered during gestation, insulin is allowed. Gestational diabetes is a condition characterized by high blood sugar (glucose) levels that is first recognized during pregnancy. The condition occurs in approximately 4% of all pregnancies and is associated with fetal macrosomia. In addition, hypertension and preeclampsia occur more commonly in women with gestational diabetes. Mitochondrial dysfunction has been reported during human pregnancy with gestational diabetes mellitus [65]. In general, some antidiabetic drugs have been related to mitochondrial damage through specific MRC impairment. For example, metformin specifically inhibits MRC complex I activity [66]. However, information regarding the consequences of insulin administration during human pregnancy and the potential mitochondrial toxicity derived from *in utero* exposure is scarce. Few works on this topic have been performed in animal models and report null effects (at least obvious) on progeny at delivery. On the contrary, indirect metabolic and mitochondrial toxicity has been reported with the development of diabetes during pregnancy due to high glucose levels that reduce fatty acid oxidation and increase triglyceride accumulation in human placenta [67], thereby causing a metabolic and energetic imbalance that may endanger embrionary development.

Nonsteroidal anti-inflammatory drugs (NSAIDs), such as aspirin or ibuprofen, are among the drugs most commonly prescribed worldwide to reduce inflammation and pain through cycloxygenase inhibition and a consequent decrease in prostaglandin production. Nonsteroidal anti-inflammatory drugs have been reported to cause an uncoupling of the oxidative phosphorylation pathway, an increase in resting state respiration, a decrease in ATP synthesis and mitochondrial membrane potential, inhibition of adenine nucleotide translocase and an alteration in mitochondrial lipid metabolic pathways with potential implications by way of the gastrointestinal damage associated with NSAID administration in non-pregnant adults [59]. However, scarce data are available on the potential effects in human pregnancies. Ibuprofen is not prescribed during pregnancy due to adverse effects on fetal circulation, but aspirin is recommended in pregnancies complicated by preeclampsia, repetitive miscarriages or previous fetal death.

Antiarrhythmic and antimalarial drugs such as quinidine have also been described to be mitotoxic agents, with quinidine being particularly associated with ATP-synthase inhibition [68]. The potential induction of oxidative stress has been associated with some antimalarial drugs [69], but little is known about their mechanisms of toxicity or adverse outcomes in pregnancy. There are few studies on the pharmacokinetics, safety and efficacy of antimalarials in human pregnancies. Several decades ago [70] one study assessed the effect of the antimalaria drug pyrimethamine in pregnancy [70,71], reporting altered embryonal hepatocytes with destructive changes in mitochondria.

In summary, although potential exposures with mitotoxic drugs during fertilization or pregnancy may be considered harmful, few studies (if any) have evaluated the clinical consequences of the use of such products in pregnant women and their potentially derived mitochondrial toxicities. Consequently, drug exposure should be minimized or avoided during pregnancy, especially when considering the above mentioned medications, except under explicit medical prescription and clinical supervision.

### 3.3. Abuse Drugs, such as Tobacco or Alcohol

It is widely known that tobacco causes serious adverse alterations in the organism. Regarding fertility, it has been described that the exposure to cigarette smoke causes antral follicle destruction and oocyte dysfunction through oxidative stress [72]. Despite a reduction in tobacco consumption during pregnancy, it is estimated that about 20–50% of pregnant women smoke during pregnancy in developed countries [18].

From a mitochondrial point of view, the carbon monoxide (CO) present in tobacco smoke binds to the heme group of the MRC complex IV, the enzyme responsible for the reduction of oxygen molecules into water, triggering a decrease in cell respiration and a consequent increase of ROS production and oxidative stress damage [73,74,75,76]. Tobacco consumption in human pregnancies has been associated with a wide range of adverse obstetric outcomes (preterm delivery, stillbirths, sudden infant death and respiratory problems during childhood), being IUGR the most frequent of these toxic manifestations [77,78]. Studies in human pregnancies have associated maternal smoking with mtDNA depletion and respiratory chain complex III deficiency in placenta [18]. In our experience [79], typical mitochondrial toxic hallmarks of smoking in pregnant women are also present in both placenta and cord blood cells of the newborn, suggesting that mitochondrial disturbances may be involved in IUGR. Such mitochondrial abnormalities due to *in utero* exposure to tobacco smoke include MRC IV inhibition, increased oxidative stress, decreased mtDNA content and raised apopototic levels which are responsible for potential adverse obstetric outcome. Placental apoptosis has been previously associated with a higher incidence of adverse obstetric outcomes, especially preeclampsia [80,81], in non-smoking women. Interestingly, IUGR may also be associated with apoptosis in smoking pregnant woman, suggesting that it may be the basis of both phenomena, regardless of the idiopathic or toxic origin.

Alcohol abuse has been widely associated with several medical and social problems and its use has been related to the intoxication of the central nervous system, impaired brain activity, poor motor coordination, and behavioral changes. Acute alcohol consumption affects carbohydrate, fat and protein metabolism. Mitochondria are essential for the conversion of acetaldehyde into acetate and the generation of increased amounts of NADH, as reviewed [82]. Therefore, during ethanol oxidation, there is an increase in the NADH/NAD^+^ ratio followed by alterations of the cellular redox state and triggering of a number of adverse effects, associated with alcohol consumption [83].

Alcohol does not bind to any tissue or plasma proteins because of its soluble nature, however, it can cross the blood brain barrier and placenta [84]. Alcohol dependence during pregnancy has been associated with a multitude of adverse obstetric outcomes and adverse effects in offspring (being the fetal alcohol syndrome the most extreme) including pre- and post-natal growth retardation, newborn microcephalia, neurologic abnormalities and intellectual disability [85]. Even moderate alcohol consumption during pregnancy carries a risk of alterations in neurodevelopment, as well as malformations and physical impairments. Early identification of chronic alcohol intake is essential for the establishment of preventive measures to diminish adverse effects among newborns [86].

Many relatively recent studies in animal models, such as rats, mice and guinea pigs [87] have been performed to determine the consequences of chronic prenatal exposure to ethanol. In these experimental studies, alcohol-induced oxidative stress and mitochondrial dysfunction has been described in placental tissue [88]. The increase in trophoblast apoptosis (associated with increased expression of pro-apoptotic proteins and decreased antiapoptotic proteins) and the increase of oxidative stress and lipid peroxidation related to gestational exposure to ethanol [88] indicate that mitochondria are the main organelle involved in all these processes. It has been reported that neuronal abnormalities found in the fetal alcohol syndrome could be due to initial damage during astrocyte development, and this syndrome has been associated with a lower number of mitochondria and presenting altered morphology [89]. Despite all these results from animal models, significant data on mitochondrial toxicity derived from *in utero* fetal exposure in human pregnancies are remarkably lacking.

### 3.4. Mitochondrial Toxicity of Poisonous Gases

There are many asphyxiating and potentially lethal gases that produce molecular damage due to its condition of mitochondrial hazards. Some of these toxic gas agents include nitric oxide (NO), cyanide (CN), hydrogen sulphide (H_2_S) or carbon monoxide (CO), some of which are responsible for severe intoxications which may induce rapid death. The severity of the symptoms and the appearance of late sequelae depend on the intensity and duration of the exposure. Interestingly, all these toxic gases present the same mitotoxic pathophysiologic mechanism of damage through the inhibition of MRC complex IV, which is ultimately responsible for oxygen consumption and cell respiration [26,90,91,92]. A consequent increase in oxidative stress steady state levels has also been described [93]. Among these gases, the most frequent intoxication is produced by CO, triggered by abnormal combustion of complex organic compounds occurring in an atmosphere lacking oxygen. This exposure can be acute (punctual) or chronic (additive). Indeed, a special case of CO intoxication is that which is characteristic of smokers because CO is one of the thousands of toxic compounds present in tobacco smoke [94]. The association of obstetric problems with mitochondrial toxicity derived from tobacco consumption during pregnancy has been previously described in the section on “Abuse drugs”.

No studies were found on mitochondrial toxicity derived from exogenous NO, CN, H_2_S or CO toxic gas exposure during pregnancy. However, a few studies did report the effects of NO and CO as endogenous physiologic factors synthesized during pregnancy. Some experimental evidence support an association of the increase of these chemical substances with the enhanced oxidative stress that takes place during pregnancy arising from increased placental mitochondrial activity and production of ROS. Myatt *et al*. reported that these ROS (NO, CO and peroxynitrite) have pronounced effects on placental function promoting vascular reactivity and trophoblast proliferation and differentiation. The description of an improved oxidative metabolism, increased endothelial NO synthase expression and NO production in human placenta after combined aerobic and resistance exercise training during the second half of pregnancy is also remarkable [95]. However, all these effects are produced by endogenous NO and CO synthesized by placenta, rather than by the exogenous gases responsible for toxic insults.

The production of ROS is part of the physiologic responses necessary at certain windows in placental development. However, excessive ROS production may occur in pathologic pregnancies, such as those complicated by preeclampsia and/or IUGR, overpowering antioxidant defenses and promoting deleterious outcomes [96]. We found no reports describing the molecular or clinical consequences of external NO or CO poisoning during pregnancy, but a potential increase in ROS generation and adverse obstetric outcome is plausible.

### 3.5. Chemical Substances such as Pesticides

Some pesticides (herbicides, insecticides or acaricides) can seriously damage the mitochondria. These compounds may induce clinical symptoms of acute intoxication but usually produce clinical manifestations after prolonged low-dose chronic exposure such as that produced by occupational contact. At present, many neurodegenerative disorders, especially Parkinson’s disease, have been associated with the toxic effect of certain chemical agents on neurons due to potential toxicant activity. Different mechanisms causing damage to the mitochondria have been described in pesticides, but the inhibition of MRC complex I enzymatic activity is one of the most common mitochondrial toxic capacities described among the most frequently used compounds (rotenone, pyridaben, fenazaquin and fenpyroximate), some of which increase ROS production (rotenone, pyridaben, paraquat) accompanied, in some cases, by the consequent development of apoptosis (paraquat and glyophosphate) [97].

For instance, blood cholinesterases and tissue carboxylesterases (CE) are sensitive indicators of exposure to environmental organophosphate pesticides (OP). In a study including healthy women living on agricultural farms, the authors studied the impact of OP exposure on placental CE activity and lipid composition during the pulverization and recess periods. Plasma and placental CE activity decreased in the pulverization period, suggesting that these pesticides reached the placenta. The cardiolipin content increased and the phosphatidylethanolamine content decreased in the light mitochondrial fraction [98], suggesting that potential detrimental toxicity may affect fetal development.

Nonetheless, further studies are needed to assess the impact of pesticides on human pregnancies due to frequent or even chronic exposure to these everyday compounds and the little information available reporting potential adverse effects on fertility or pregnancy outcomes.

## 4. Discussion

Several studies have suggested that the mitochondria are critical for successful fertilization and fetal development [14]. For instance, mitochondrial dysfunction has been associated with reproductive outcome since their function influences the viability of both sperm and oocytes. Accordingly, a low mtDNA content in both males [99,100] and females [4,44] has been related to infertility. In addition, mutations in the mtDNA genome have also been described in spermatozoa with declined motility and fertility [101].

In general, disorders of mitochondrial function in oocytes may cause reproductive failure. Mitochondrial defects in oocytes can eventually lead to cell dysfunction and infertility and mitochondrial content has been demonstrated to reflect oocyte viability and fertilization outcome [4]. Both Reynier [100] and Santos [4] established an association between the mtDNA content and fertilization. The latter study suggested that mtDNA content could be a marker of oocyte quality and fertility. Other studies have suggested that a low mtDNA content is associated with the impaired oocyte quality observed in ovarian insufficiency [102].

Accordingly, adequate mitochondrial and mtDNA content in both male and female gametes is intrinsic for successful fertility. Nonetheless, once fertilization is achieved, mitochondrial function is still essential for fetal development and a favorable obstetric outcome. Some studies have analyzed the implication of mtDNA levels in fetal growth. Gemma *et al*. reported that newborns with abnormally low and high birth weight present less mtDNA content in the umbilical cord [79,103]. More recently, other authors corroborated these results in cord blood leucocytes of neonates with reduced body mass [103,104], suggesting that appropriate mtDNA levels are essential for appropriate birth weight development. Intrauterine growth restriction and pregnancy hypertensive disorders such as preeclampsia associated with IUGR share a common placental phenotype called “placental insufficiency”, originating early in pregnancy when the availability of high energy output is required. This period is characterized by decidual trophoblast invasion and intense cellular growth, replication and differentiation. Since a huge energetic production is required during gestation, the mitochondria may play a crucial role in this process, being the main energetic producer in the cell.

Mitochondrial disturbances can arise from both inherited diseases and exposure to toxic substances. Genetically inherited diseases can be suspected and avoided through preconceptional genetic counseling. However, mitochondrial toxicities can be acquired by healthy pregnant women exposed to mitochondrial toxic compounds during gestation, with consequences for affected newborns similar to those of mitochondriopathies of genetic origin. Dozens of compounds in our everyday life are toxic for mitochondria, including some found in daily clinical practice. A large number of studies have reported the toxic adverse effects of these mitochondrial poisons in non-pregnant subjects. However, only a few studies have analyzed the effects derived from *in utero* exposure. Indeed, there are very scarce data regarding the use of antipsychotics, antibiotics, antidiabetics, anaesthetics, antimalarials or fungicides, among others, during gestation.

It is unclear whether the lack of information related to these factors is related to the inability to undertake designated studies (exclusively observational or case-reports are available) or if the mitochondrial toxicity in pregnant women has minimal consequences on obstetric outcomes. On the contrary, the number of studies reporting information about HIV, ARV and tobacco exposure during gestation was higher, probably due to a larger number of pregnancies affected by such exposures.

The negative mitochondrial and clinical effects of ARV have been focused on HIV-human pregnancies with some controversial results [45,50]. However, most studies associated ARV-derived mitochondrial toxicity with significant mtDNA depletion and mitochondrial dysfunction in maternal, fetal and/or placental tissues, usually demonstrating a strong, significant and positive correlation in materno-fetal mitochondrial lesion. Antiretroviral drugs are able to cross the placental barrier. Our group reached similar conclusions regarding the mitochondrial toxicity observed in smoking pregnant women: newborns exposed to tobacco *in utero* showed a similar mitochondrial toxicity to that of their smoking mothers [79]. Again, substances derived from tobacco which are toxic to the mitochondria (such as CO) present transplacental activity and may reach the fetus in this crucial stage of development. Transplacental mitochondrial toxicities have also been demonstrated in pesticide exposure [98], once again demonstrating the strong relationship of materno-fetal toxicities.

The adverse clinical effects of these mitochondrial toxicities have been reported both in exposed mothers and fetuses along gestation. Increased adverse obstetric outcomes are frequent in these pregnancies. Maternal clinical and mitochondrial recovery is feasible once the toxic exposure is discontinued (whenever possible). However, during fetal development a multitude of physiologic responses take place in mitochondrially endangered fetuses to overcome the energetic imbalance and/or oxidative and apoptotic insult. The homeostatic mechanisms responsible for fetal remodeling of the adaptative response cannot be reversed once the fetuses are born and the toxic exposure is disrupted (if possible). All this molecular and cell fetal remodeling derived from exposure to mitochondrial toxic compounds *in utero* may have long term consequences of unknown severity.

There is currently no therapeutic option available for mitochondrial pathologies or for their potential transmission. Selenium supplementation has been proposed as a protector mechanism of trophoblast cells from oxidative stress [105]. However, to date, genetic counseling and preimplantacional diagnosis are the best therapeutic options to avoid the transmission of mitochondrial diseases. Future IVF strategies including the use of restriction enzymes to decrease the number of mutated mtDNA molecules in the maternal oocyte or mitochondrial replacement techniques (through *Spindel* and *Pronuclear Transfer*) may prevent the transmission of mitochondrial disease (in the former by using three reproductive cells; the extra oocyte is necessary to provide healthy mitochondria) [106]. However, when these therapeutic options become available they will probably be devoted to avoiding transmission of inherited mitochondrial diseases. Nonetheless, despite having similar molecular and clinical consequences, mitochondrial diseases acquired by toxic exposure to any mitochondrial poison, cannot, at present, be treated or prevented.

Consequently, further studies are needed to determine the fetal and future neonatal consequences of exposure to mitochondrial toxic agents *in utero* which are frequently present in our everyday life. Currently, it is best to avoid exposure to all the above mentioned toxic mitochondrial hazards. Nevertheless, to prevent damage, in case of therapeutic or accidental exposure, further research is required to find compounds which are less harmful to the mitochondria and to establish or translate biomarkers into clinical practice to follow mitochondrial lesions in pregnant women.

## 5. Conclusions

Mitochondrial toxicity derived from exposure to toxic agents can potentially involve much more severe consequences in the context of pregnancy than in adulthood as they may condition fetal remodeling, physiologic alterations and irreversible changes in the life of the developing individual.

Further studies are needed to elucidate the impact of mitochondrial toxic substances during the fertile stage and especially during pregnancy in humans in order to understand the precise molecular mechanisms leading to adverse clinical events.

As the current treatments are symptomatic and supportive rather than therapeutic, the prevention of acquired mitochondrial diseases and exposure to mitochondrial toxicity is, to date, the best prophylactic therapeutic option of choice.

Since prevention is not always possible in therapeutic or accidental exposures, there is the crucial need to find substances which are less toxic to the mitochondria, to search for biomarkers which facilitate monitoring during pregnancy and to assess the risk-benefit imbalance in cases of treatment prescription. Indeed, some biomarkers have already been developed and should be applied to the clinical field.

## References

[1] Scheffler I.E. (2010). Mitochondria.

[2] Dyall S.D., Brown M.T., Johnson P.J. (2004). Ancient invasions: From endosymbionts to organelles. Science.

[3] Anderson S., Bankier A.T., Barrell B.G., de Bruijn M.H., Coulson A.R., Drouin J., Eperon I.C., Nierlich D.P., Roe B.A., Sanger F. (1981). Sequence and organization of the human mitochondrial genome. Nature.

[4] Santos T.A., El Shourbagy S., St John J.C. (2006). Mitochondrial content reflects oocyte variability and fertilization outcome. Fertil. Steril..

[5] Jacobs L.J., de Wert G., Geraedts J.P., de Coo I.F., Smeets H.J. (2006). The transmission of OXPHOS disease and methods to prevent this. Hum. Reprod. Update.

[6] Shoubridge E.A., Wai T. (2007). Mitochondrial DNA and the mammalian oocyte. Curr. Top. Dev. Biol..

[7] Marchington D.R., Macaulay V., Hartshorne G.M., Barlow D., Poulton J. (1998). Evidence from human oocytes for a genetic bottleneck in an mtDNA disease. Am. J. Hum. Genet..

[8] Hardy K., Spanos S., Becker D., Iannelli P., Winston R.M., Stark J. (2001). From cell death to embryo arrest: Mathematical models of human preimplantation embryo development. Proc. Natl. Acad. Sci. USA.

[9] Wells D., Delhanty J.D. (2000). Comprehensive chromosomal analysis of human preimplantation embryos using whole genome amplification and single cell comparative genomic hybridization. Mol. Hum. Reprod..

[10] Moor R.M., Dai Y., Lee C., Fulka J. (1998). Oocyte maturation and embryonic failure. Hum. Reprod. Update.

[11] Artley J.K., Braude P.R., Cooper P. (1993). Vaginal squamous cells in follicular aspirates following transvaginal puncture. Hum. Reprod..

[12] Bavister B.D. (1995). Culture of preimplantation embryos: Facts and artifacts. Hum. Reprod. Update.

[13] Bain N.T., Madan P., Betts D.H. (2011). The early embryo response to intracellular reactive oxygen species is developmentally regulated. Reprod. Fertil. Dev..

[14] Cohen J., Scott R., Schimmel T., Levron J., Willadsen S. (1997). Birth of infant after transfer of anucleate donor oocyte cytoplasm into recipient eggs. Lancet.

[15] Taylor R.W., Turnbull D.M. (2005). Mitochondrial DNA mutations in human disease. Nat. Rev. Genet..

[16] Andreu A.L., Gonzalo-Sanz R. (2004). Mitochondrial disorders: A classification for the 21st century. Neurology..

[17] Mando C., de Palma C., Stampalija T., Anelli G.M., Figus M., Novielli C., Parisi F., Clementi E., Ferrazzi E., Cetin I. (2014). Placental mitochondrial content and function in intrauterine growth restriction and preeclampsia. Am. J. Physiol. Endocrinol. Metab..

[18] Bouhours-Nouet N., May-Panloup P., Coutant R., de Casson F.B., Descamps P., Douay O., Reynier P., Ritz P., Malthièry Y., Simard G. (2005). Maternal smoking is associated with mitochondrial DNA depletion and respiratory chain complex III deficiency in placenta. Am. J. Physiol. Endocrinol. Metab..

[19] Garrabou G., Soriano A., Lopez S., Guallar J.P., Giralt M., Villarroya F., Martínez J.A., Casademont J., Cardellach F., Mensa J. (2007). Reversible inhibition of mitochondrial protein synthesis during linezolid-related hyperlactatemia. Antimicrob. Agents Chemother..

[20] Finsterer J. (2010). Treatment of mitochondrial disorders. Eur. J. Paediatr. Neurol..

[21] Berkley E.M., Leslie K.K., Arora S., Qualls C., Dunkelberg J.C. (2008). Chronic hepatitis C in pregnancy. Obstet. Gynecol..

[22] Ezechi O.C., Gab-Okafor C.V., Oladele D.A., Kalejaiye O.O., Oke B.O., Ohwodo H.O., Adu R.A., Ekama S.O., Musa Z., Onwujekwe D.I. (2013). Pregnancy, obstetric and neonatal outcomes in HIV positive Nigerian women. Afr. J. Reprod. Health.

[23] Yen H.H., Shih K.L., Lin T.T., Su W.W., Soon M.S., Liu C.S. (2012). Decreased mitochondrial deoxyribonucleic acid and increased oxidative damage in chronic hepatitis C. World J. Gastroenterol..

[24] Ferri K.F., Jacotot E., Blanco J., Este J.A., Kroemer G. (2000). Mitochondrial control of cell death induced by HIV-1-encoded proteins. Ann. N. Y. Acad. Sci..

[25] Cote H.C., Brumme Z.L., Craib K.J., Alexander C.S., Wynhoven B., Ting L., Wong H., Harris M., Harrigan P.R., O’Shaughnessy M.V. (2002). Changes in mitochondrial DNA as a marker of nucleoside toxicity in HIV-infected patients. N. Engl. J. Med..

[26] Miro O., Alonso J.R., Lopez S., Beato A., Casademont J., Cardellach F. (2004). *Ex vivo* analysis of mitochondrial function in patients attended in an emergency department due to carbon monoxide poisoning. Med. Clin. Barc..

[27] Giacomet V., Vigano A., Erba P., Nannini P., Pisanelli S., Zanchetta N., Brambilla T., Ramponi G., Zuccotti G.V. (2014). Unexpected vertical transmission of HIV infection. Eur. J. Pediatr..

[28] Townsend C.L., Cortina-Borja M., Peckham C.S., de Ruiter A., Lyall H., Tookey P.A. (2008). Low rates of mother-to-child transmission of HIV following effective pregnancy interventions in the United Kingdom and Ireland, 2000–2006. AIDS.

[29] Ciaranello A.L., Seage G.R., Freedberg K.A., Weinstein M.C., Lockman S., Walensky R.P. (2008). Antiretroviral drugs for preventing mother-to-child transmission of HIV in sub-Saharan Africa: Balancing efficacy and infant toxicity. AIDS.

[30] Zylla D., Li Y., Bergenstal E., Merrill J.D., Douglas S.D., Mooney K., Guo C.J., Song L., Ho W.Z. (2003). CCR5 expression and beta-chemokine production during placental neonatal monocyte differentiation. Pediatr. Res..

[31] Tuomala R.E., Watts D.H., Li D., Vajaranant M., Pitt J., Hammill H., Landesman S., Zorrilla C., Thompson B., Women and Infants Transmission Study (2005). Improved obstetric outcomes and few maternal toxicities are associated with antiretroviral therapy, including highly active antiretroviral therapy during pregnancy. J. Acquir. Immune Defic. Syndr..

[32] Tuomala R.E., Shapiro D.E., Mofenson L.M., Bryson Y., Culnane M., Hughes M.D., O’Sullivan M.J., Scott G., Stek A.M., Wara D. (2002). Antiretroviral therapy during pregnancy and the risk of an adverse outcome. N. Engl. J. Med..

[33] Lambert J.S., Watts D.H., Mofenson L., Stiehm E.R., Harris D.R., Bethel J., Whitehouse J., Jimenez E., Gandia J., Scott G. (2000). Risk factors for preterm birth, low birth weight, and intrauterine growth retardation in infants born to HIV-infected pregnant women receiving zidovudine. Pediatric AIDS Clinical Trials Group 185 Team. AIDS.

[34] Brocklehurst P., French R. (1998). The association between maternal HIV infection and perinatal outcome: A systematic review of the literature and meta-analysis. Br. J. Obstet. Gynaecol..

[35] Thorne C., Patel D., Newell M.L. (2004). Increased risk of adverse pregnancy outcomes in HIV-infected women treated with highly active antiretroviral therapy in Europe. AIDS.

[36] Kirmse B., Baumgart S., Rakhmanina N. (2013). Metabolic and mitochondrial effects of antiretroviral drug exposure in pregnancy and postpartum: Implications for fetal and future health. Semin. Fetal Neonatal Med..

[37] Suy A., Martinez E., Coll O., Lonca M., Palacio M., de Lazzari E., Larrousse M., Milinkovic A., Hernández S., Blanco J.L. (2006). Increased risk of pre-eclampsia and fetal death in HIV-infected pregnant women receiving highly active antiretroviral therapy. AIDS.

[38] Wimalasundera R.C., Larbalestier N., Smith J.H., de Ruiter A., McGThom S.A., Hughes A.D., Poulter N., Regan L., Taylor G.P. (2002). Pre-eclampsia, antiretroviral therapy, and immune reconstitution. Lancet.

[39] Rudin C., Spaenhauer A., Keiser O., Rickenbach M., Kind C., Aebi-Popp K., Brinkhof M.W., Swiss HIV Cohort Study (SHCS), Swiss Mother & Child HIV Cohort Study (MoCHiV) (2011). Antiretroviral therapy during pregnancy and premature birth: Analysis of Swiss data. HIV Med..

[40] Haeri S., Shauer M., Dale M., Leslie J., Baker A.M., Saddlemire S., Boggess K. (2009). Obstetric and newborn infant outcomes in human immunodeficiency virus-infected women who receive highly active antiretroviral therapy. Am. J. Obstet. Gynecol..

[41] Townsend C.L., Cortina-Borja M., Peckham C.S., Tookey P.A. (2007). Antiretroviral therapy and premature delivery in diagnosed HIV-infected women in the United Kingdom and Ireland. AIDS.

[42] Apostolova N., Blas-Garcia A., Esplugues J.V. (2011). Mitochondrial toxicity in HAART: An overview of *in vitro* evidence. Curr. Pharm. Des..

[43] Kushnir V.A., Lewis W. (2011). Human immunodeficiency virus/acquired immunodeficiency syndrome and infertility: Emerging problems in the era of highly active antiretrovirals. Fertil. Steril..

[44] Lopez S., Coll O., Durban M., Hernandez S., Vidal R., Suy A., Morén C., Casademont J., Cardellach F., Mataró D. (2008). Mitochondrial DNA depletion in oocytes of HIV-infected antiretroviral-treated infertile women. Antivir. Ther..

[45] Hernandez S., Moren C., Lopez M., Coll O., Cardellach F., Gratacos E, Miró O., Garrabou G. (2012). Perinatal outcomes, mitochondrial toxicity and apoptosis in HIV-treated pregnant women and in-utero-exposed newborn. AIDS.

[46] Divi R.L., Walker V.E., Wade N.A., Nagashima K., Seilkop S.K., Adams M.E., Nesel C.J., O’Neill J.P., Abrams E.J., Poirier M.C. (2004). Mitochondrial damage and DNA depletion in cord blood and umbilical cord from infants exposed *in utero* to Combivir. AIDS.

[47] Aldrovandi G.M., Chu C., Shearer W.T., Li D., Walter J., Thompson B., McIntosh K., Foca M., Meyer W.A., Ha B.F. (2009). Antiretroviral exposure and lymphocyte mtDNA content among uninfected infants of HIV-1-infected women. Pediatrics.

[48] Poirier M.C., Divi R.L., Al-Harthi L., Olivero O.A., Nguyen V., Walker B., Landay A.L., Walker V.E., Charurat M., Blattner W.A. (2003). Long-term mitochondrial toxicity in HIV-uninfected infants born to HIV-infected mothers. J. Acquir. Immune Defic. Syndr..

[49] Shiramizu B., Shikuma K.M., Kamemoto L., Gerschenson M., Erdem G., Pinti M., Cossarizza A., Shikuma C. (2003). Placenta and cord blood mitochondrial DNA toxicity in HIV-infected women receiving nucleoside reverse transcriptase inhibitors during pregnancy. J. Acquir. Immune Defic. Syndr..

[50] Ross A.C., Leong T., Avery A., Castillo-Duran M., Bonilla H., Lebrecht D., Walker U.A., Storer N., Labbato D., Khaitan A. (2012). Effects of *in utero* antiretroviral exposure on mitochondrial DNA levels, mitochondrial function and oxidative stress. HIV Med..

[51] Ramsay R.R., Singer T.P. (1992). Relation of superoxide generation and lipid peroxidation to the inhibition of NADH-Q oxidoreductase by rotenone, piericidin A, and MPP+. Biochem. Biophys. Res. Commun..

[52] Hanley P.J., Ray J., Brandt U., Daut J. (2002). Halothane, isoflurane and sevoflurane inhibit NADH: Ubiquinone oxidoreductase (complex I) of cardiac mitochondria. J. Physiol..

[53] Short T.G., Young Y. (2003). Toxicity of intravenous anaesthetics. Best Pract. Res. Clin. Anaesthesiol..

[54] Fau D., Eugene D., Berson A., Letteron P., Fromenty B., Fisch C., Pessayre D. (1994). Toxicity of the antiandrogen flutamide in isolated rat hepatocytes. J. Pharmacol. Exp. Ther..

[55] Cardoso C.M., Custodio J.B., Almeida L.M., Moreno A.J. (2001). Mechanisms of the deleterious effects of tamoxifen on mitochondrial respiration rate and phosphorylation efficiency. Toxicol. Appl. Pharmacol..

[56] Davoudi M., Kallijarvi J., Marjavaara S., Kotarsky H., Hansson E., Leveen P., Fellman V. (2014). A mouse model of mitochondrial complex III dysfunction induced by myxothiazol. Biochem. Biophys. Res. Commun..

[57] Brunmair B., Lest A., Staniek K., Gras F., Scharf N., Roden M., Nohl H., Waldhäusl W., Fürnsinn C. (2004). Fenofibrate impairs rat mitochondrial function by inhibition of respiratory complex I. J. Pharmacol. Exp. Ther..

[58] El-Schahawi M., Lopez de Munain A., Sarrazin A.M., Shanske A.L., Basirico M., Shanske S., DiMauro S. (1997). Two large Spanish pedigrees with nonsyndromic sensorineural deafness and the mtDNA mutation at nt 1555 in the 12s rRNA gene: Evidence of heteroplasmy. Neurology.

[59] Szewczyk A., Wojtczak L. (2002). Mitochondria as a pharmacological target. Pharmacol. Rev..

[60] Modifier Factors Influencing the Phenotypic Manifestation of the Deafness Associated Mitochondrial DNA Mutations. http://www.bioportfolio.com/resources/pmarticle/167525/Modifier-factors-influencing-the-phenotypic-manifestation-of-the-deafness-associated-mitochondrial-DNA.html.

[61] Bressler A.M., Zimmer S.M., Gilmore J.L., Somani J. (2004). Peripheral neuropathy associated with prolonged use of linezolid. Lancet Infect. Dis..

[62] Del Pozo J.L., Fernandez-Ros N., Saez E., Herrero J.I., Yuste J.R., Banales J.M. (2014). Linezolid-induced lactic acidosis in two liver transplant patients with the mitochondrial DNA A2706G polymorphism. Antimicrob. Agents Chemother..

[63] Zhou Z.Y., Zhao X.Q., Shan B.Z., Zhu J., Zhang X., Tian Q.F., Chen D.F., Jia T.H. (2014). Efficacy and safety of linezolid in treating gram-positive bacterial infection in the elderly: A retrospective study. Indian J. Microbiol..

[64] Casademont J., Garrabou G., Miro O., Lopez S., Pons A., Bernardo M., Cardellach F. (2007). Neuroleptic treatment effect on mitochondrial electron transport chain: Peripheral blood mononuclear cells analysis in psychotic patients. J. Clin. Psychopharmacol..

[65] Boyle K.E., Newsom S.A., Janssen R.C., Lappas M., Friedman J.E. (2013). Skeletal muscle MnSOD, mitochondrial complex II, and SIRT3 enzyme activities are decreased in maternal obesity during human pregnancy and gestational diabetes mellitus. J. Clin. Endocrinol. Metab..

[66] Brunmair B., Staniek K., Gras F., Scharf N., Althaym A., Clara R., Roden M., Gnaiger E., Nohl H., Waldhäusl W. (2004). Thiazolidinediones, like metformin, inhibit respiratory complex I: A common mechanism contributing to their antidiabetic actions?. Diabetes.

[67] Visiedo F., Bugatto F., Sanchez V., Cozar-Castellano I., Bartha J.L., Perdomo G. (2013). High glucose levels reduce fatty acid oxidation and increase triglyceride accumulation in human placenta. Am. J. Physiol. Endocrinol. Metab..

[68] Almotrefi A.A. (1993). Effects of class I antiarrhythmic drugs on mitochondrial ATPase activity in guinea pig heart preparations. Gen. Pharmacol..

[69] Alberti A., Macciantelli D., Marconi G. (2004). Free radicals formed by addition of antimalaric artemisinin (Qinghaosu, QHS) to human serum: An ESR-spin trapping investigation. Res. Chem. Intermed..

[70] Bariliak I.R., Kalinovskaia L.P. (1979). Histochemical and ultrastructural characteristics of embryonal hepatocytes exposed to chloridin (pyrimethamine). Tsitol. Genet..

[71] Nosten F., McGready R., d’Alessandro U., Bonell A., Verhoeff F., Menendez C., Mutabingwa T., Brabin B. (2006). Antimalarial drugs in pregnancy: A review. Curr. Drug Saf..

[72] Sobinoff A.P., Beckett E.L., Jarnicki A.G., Sutherland J.M., McCluskey A., Hansbro P.M., McLaughlin E.A. (2013). Scrambled and fried: Cigarette smoke exposure causes antral follicle destruction and oocyte dysfunction through oxidative stress. Toxicol. Appl. Pharmacol..

[73] Piantadosi C.A., Carraway M.S., Suliman H.B. (2006). Carbon monoxide, oxidative stress, and mitochondrial permeability pore transition. Free Radic. Biol. Med..

[74] Cardellach F., Alonso J.R., Lopez S., Casademont J., Miro O. (2003). Effect of smoking cessation on mitochondrial respiratory chain function. J. Toxicol. Clin. Toxicol..

[75] Queiroga C.S., Almeida A.S., Vieira H.L. (2012). Carbon monoxide targeting mitochondria. Biochem. Res. Int..

[76] Alonso J.R., Cardellach F., Lopez S., Casademont J., Miro O. (2003). Carbon monoxide specifically inhibits cytochrome c oxidase of human mitochondrial respiratory chain. Pharmacol. Toxicol..

[77] Mitchell E.A., Ford R.P., Stewart A.W., Taylor B.J., Becroft D.M., Thompson J.M., Scragg R., Hassall I.B., Barry D.M., Allen E.M. (1993). Smoking and the sudden infant death syndrome. Pediatrics.

[78] Pattenden S., Antova T., Neuberger M., Nikiforov B., De Sario M., Grize L., Heinrich J., Hruba F., Janssen N., Luttmann-Gibson H. (2006). Parental smoking and children’s respiratory health: Independent effects of prenatal and postnatal exposure. Tob. Control..

[79] Garrabou G.H.A., Catalán M., Morén C., Tobías E., Córdoba S., López M., Figueras F., Grau J.M., Cardellach F. (2014). Molecular basis of reduced birth weight in smoking pregnant women: Mitochondrial dysfunction and apoptosis. Addict. Biol..

[80] Cali U., Cavkaytar S., Sirvan L., Danisman N. (2013). Placental apoptosis in preeclampsia, intrauterine growth retardation, and HELLP syndrome: An immunohistochemical study with caspase-3 and bcl-2. Clin. Exp. Obstet. Gynecol..

[81] Kim Y.N., Kim H.K., Warda M., Kim N., Park W.S., Prince Adel B., Jeong D.H., Lee D.S., Kim K.T., Han J. (2007). Toward a better understanding of preeclampsia: Comparative proteomic analysis of preeclamptic placentas. Proteomics Clin. Appl..

[82] Manzo-Avalos S., Saavedra-Molina A. (2010). Cellular and mitochondrial effects of alcohol consumption. Int. J. Environ. Res. Public Health.

[83] Norberg A., Jones A.W., Hahn R.G., Gabrielsson J.L. (2003). Role of variability in explaining ethanol pharmacokinetics: Research and forensic applications. Clin. Pharmacokinet.

[84] Streissguth A.P., Landesman-Dwyer S., Martin J.C., Smith D.W. (1980). Teratogenic effects of alcohol in humans and laboratory animals. Science.

[85] Romera Modamio G., Fernandez Lopez A., Jordan Garcia Y., Pastor Gomez A., Rodriguez Miguelez J.M., Botet Mussons F., Figueras Aloy J. (1997). Alcoholic embryofetopathy. Neonatal case reports for the past twelve years. An. Esp. Pediatr..

[86] Bana A., Tabernero M.J., Perez-Munuzuri A., Lopez-Suarez O., Dosil S., Cabarcos P., Bermejo A., Fraga J.M., Couce M.L. (2014). Prenatal alcohol exposure and its repercussion on newborns. J. Neonatal Perinatal Med..

[87] Green C.R., Watts L.T., Kobus S.M., Henderson G.I., Reynolds J.N., Brien J.F. (2006). Effects of chronic prenatal ethanol exposure on mitochondrial glutathione and 8-iso-prostaglandin F2alpha concentrations in the hippocampus of the perinatal guinea pig. Reprod. Fertil. Dev..

[88] Gundogan F., Elwood G., Mark P., Feijoo A., Longato L., Tong M., de la Monte S.M. (2010). Ethanol-induced oxidative stress and mitochondrial dysfunction in rat placenta: Relevance to pregnancy loss. Alcohol. Clin. Exp. Res..

[89] Mayordomo F., Renau-Piqueras J., Megias L., Guerri C., Iborra F.J., Azorin I., Ledig M. (1992). Cytochemical and stereological analysis of rat cortical astrocytes during development in primary culture. Effect of prenatal exposure to ethanol. Int. J. Dev. Biol..

[90] Cardellach F., Miro O., Casademont J. (2003). Hyperbaric oxygen for acute carbon monoxide poisoning. N. Engl. J. Med..

[91] Garrabou G., Inoriza J.M., Moren C., Oliu G., Miro O., Marti M.J., Cardellach F. (2011). Mitochondrial injury in human acute carbon monoxide poisoning: The effect of oxygen treatment. J. Environ. Sci. Health C Environ. Carcinog. Ecotoxicol. Rev..

[92] Hyperbaric Oxygen Therapy for Carbon Monoxide Poisoning. http://www.downloads.imune.net/journals/1987%20Updated%20Studies%20on%20Hyper-Baric%20Oxygen/pdf/Hyperbaric%20oxygen%20therapy%20for%20carbon%20monoxide%20poisoning.pdf.

[93] Akyol S., Erdogan S., Idiz N., Celik S., Kaya M., Ucar F., Dane S., Akyol O. (2014). The role of reactive oxygen species and oxidative stress in carbon monoxide toxicity: An in-depth analysis. Redox Rep..

[94] Harris I.S., Blaser H., Moreno J., Treloar A.E., Gorrini C., Sasaki M., Mason J.M., Knobbe C.B., Rufini A., Hallé M. (2013). PTPN12 promotes resistance to oxidative stress and supports tumorigenesis by regulating FOXO signaling. Oncogene.

[95] Ramirez-Velez R., Bustamante J., Czerniczyniec A., Aguilar de Plata A.C., Lores-Arnaiz S. (2013). Effect of exercise training on eNOS expression, NO production and oxygen metabolism in human placenta. PLoS One.

[96] Myatt L., Cui X. (2004). Oxidative stress in the placenta. Histochem. Cell Biol..

[97] Benachour N., Seralini G.E. (2009). Glyphosate formulations induce apoptosis and necrosis in human umbilical, embryonic, and placental cells. Chem. Res. Toxicol..

[98] Vera B., Santa Cruz S., Magnarelli G. (2012). Plasma cholinesterase and carboxylesterase activities and nuclear and mitochondrial lipid composition of human placenta associated with maternal exposure to pesticides. Reprod. Toxicol..

[99] Kao S.H., Chao H.T., Liu H.W., Liao T.L., Wei Y.H. (2004). Sperm mitochondrial DNA depletion in men with asthenospermia. Fertil. Steril..

[100] Reynier P., May-Panloup P., Chretien M.F., Morgan C.J., Jean M., Savagner F., Barrière P., Malthièry Y. (2001). Mitochondrial DNA content affects the fertilizability of human oocytes. Mol. Hum. Reprod..

[101] Kao S.H., Chao H.T., Wei Y.H. (1998). Multiple deletions of mitochondrial DNA are associated with the decline of motility and fertility of human spermatozoa. Mol. Hum. Reprod..

[102] May-Panloup P., Chretien M.F., Jacques C., Vasseur C., Malthiery Y., Reynier P. (2005). Low oocyte mitochondrial DNA content in ovarian insufficiency. Hum. Reprod..

[103] Gemma C., Sookoian S., Alvarinas J., Garcia S.I., Quintana L., Kanevsky D., González C.D., Pirola C.J. (2006). Mitochondrial DNA depletion in small- and large-for-gestational-age newborns. Obesity Silver Spring.

[104] Pejznochova M., Tesarova M., Honzik T., Hansikova H., Magner M., Zeman J. (2008). The developmental changes in mitochondrial DNA content per cell in human cord blood leukocytes during gestation. Physiol. Res..

[105] Khera A., Vanderlelie J.J., Perkins A.V. (2013). Selenium supplementation protects trophoblast cells from mitochondrial oxidative stress. Placenta.

[106] Amato P., Tachibana M., Sparman M., Mitalipov S. (2014). Three-parent *in vitro* fertilization: Gene replacement for the prevention of inherited mitochondrial diseases. Fertil. Steril..

