# *Addendum:* Morén, C.; Hernández, S.; Guitart-Mampel, M.; Garrabou, G. Mitochondrial Toxicity in Human Pregnancy: An Update on Clinical and Experimental Approaches in the Last 10 Years. *Int. J. Environ. Res. Public Health* 2014, *11*, 9897–9918

**DOI:** 10.3390/ijerph13111108

**Published:** 2016-11-08

**Authors:** Constanza Morén, Sandra Hernández, Mariona Guitart-Mampel, Glòria Garrabou

**Affiliations:** 1 Muscle Research and Mitochondrial Function Laboratory, Cellex-IDIBAPS-Faculty of Medicine-University of Barcelona, Internal Medicine Service-Hospital Clínic of Barcelona, Barcelona 08036, Spain; garrabou@clinic.ub.es; 2 Centro de Investigación Biomédica en Red (CIBER) de Enfermedades Raras, CIBERER, Valencia 46010, Spain; ashernan@clinic.ub.es; 3 Materno-Fetal Medicine Department, Clinical Institute of Gynaecology, Obstetrics and Neonatology, Barcelona 08025, Spain

The authors wish to update the Acknowledgments Section in their paper published in the *International Journal of Environmental Research and Public Health* [1], doi:10.3390/ijerph110909897, website: http://www.mdpi.com/1660-4601/11/9/9897.
